# Shared Decision‐Making Theories, Conceptual Models, and Frameworks Within Oral Healthcare: A Theory Analysis

**DOI:** 10.1111/idh.70074

**Published:** 2026-04-16

**Authors:** Jessica Persson Kylén, Dawn Stacey, Inger Wårdh, Krystina B. Lewis

**Affiliations:** ^1^ Department of Health Sciences Karlstad University Karlstad Sweden; ^2^ Centre for a Dementia‐Friendly Society With A Person‐Centred Mindset, Institute of Health and Care Science The Sahlgrenska Academy, Gothenburg University Sweden; ^3^ Research and Development Department Public Dental Service, Region Västra Götaland Vänersborg Sweden; ^4^ School of Nursing, Faculty of Health Sciences University of Ottawa Canada; ^5^ Centre for Implementation Research Ottawa Hospital Research Institute Canada; ^6^ Department of Dental Medicine Karolinska Institute Huddinge Sweden; ^7^ Academic Centre for Geriatric Dentistry Stockholm Sweden

**Keywords:** conceptual models, oral health, person‐centred care, shared decision making, theory analysis

## Abstract

**Objective:**

To appraise the quality of theories, conceptual models, and theoretical frameworks about SDM for oral healthcare practices.

**Background:**

Shared decision‐making (SDM) is crucial to person‐centred care. Yet little is known about how (and if) patient engagement in SDM is conceptualised in oral healthcare.

**Methods:**

We conducted a theory analysis of theories, conceptual models, and frameworks. We searched Medline, Scopus, and CINAHL. Eligible theories/conceptual models/frameworks had to specifically focus on SDM in oral healthcare. Eligible papers were analysed using Walker and Avant's theory analysis steps and mapped onto the 8 core elements of SDM.

**Main Findings:**

Of 195 citations, two conceptual models specific to oral healthcare were identified. The SDM System on Dental Restorations (*SDMS‐DR*; South Korea) focuses on decisions between dentists and patients about restorative treatment. The Four + one Habits Model for dental visits (*4 + 1 HB*; Norway) is an approach for SDM across oral healthcare decisions, more broadly. Both conceptual models focus on the patient‐professional encounter and met 4 (SDMS‐DR) and 6 (4 + 1HB) of the 8 core elements of SDM. The SDMS‐DR provided no reference to the patients' expertise. The 4 + 1HB presented SDM as a standardised linear process rather than a dynamic process. Neither has been empirically tested, and they do not acknowledge the broader healthcare system within which the encounters take place.

**Conclusion:**

We identified two conceptual models that incorporated some elements of SDM for oral healthcare. Neither was evaluated, and both focus exclusively on the patient‐oral healthcare professional encounter, without consideration of intersectional, multidisciplinary healthcare services.

## 1 Introduction

Oral health is essential for quality of life and overall health, impacting individuals' physical, social, and mental well‐being [1, 2, 3, 4]. Oral diseases are the most widespread noncommunicable diseases in the world and a significant public health concern. They are associated with malnutrition, diabetes, respiratory and cardiovascular diseases [5, 6, 7, 8]. In a recent review in *The Lancet*, Watt et al. [9] called for the oral healthcare domain to further integrate with general healthcare systems, placing greater emphasis on holistic, person‐centred care. A person‐centred care approach treats each person receiving care as unique, considering their personal preferences, vulnerabilities, resources, and overall health capabilities beyond their specific oral health condition [10].

Shared decision‐making (SDM) has been described as the crux of person‐centred care [11]. It is defined as a process by which a choice is made by the patient together with one or more healthcare professionals [12, 13]. The goal of SDM is to make informed, quality decisions by considering the best evidence on risks and benefits, along with patients' preferences and values [14]. SDM improves affective‐cognitive patient outcomes, including knowledge and satisfaction with the decision‐making process [15].

People seeking oral healthcare face decisions about promoting, improving, or maintaining oral health. Some people find decisions about getting dental implants, periodontal surgery, or orthodontic treatments difficult [16, 17]. Other difficult decisions include tooth extractions and dentures. Patients seeking healthcare services want information regarding their options and want to be actively engaged in making these decisions [18, 19]. Oral healthcare guidelines stress the importance of decision‐making and treatment planning for various conditions [17, 20, 21]. However, perceived barriers in oral healthcare decision‐making include a lack of time and limited knowledge among professionals on presenting and discussing multiple treatment options with patients [22], while a recent Swedish study found that nurses and dental hygienists agreed that involving older adults in oral healthcare planning at home was crucial for their health [23]. However, nurses and dental hygienists acknowledged not being used to conducting healthcare planning and decisions together. Hence, there is a need to explore the theoretical underpinnings within oral healthcare to guide oral healthcare professionals in shared decision‐making to achieve person‐centred care.

Theoretical foundations aid in conceptualisation, understanding, and application in education, research, and clinical practice [24]. Theory analysis provides a robust method for systematically evaluating existing theories, models, and frameworks [24]. First, it provides deeper insights into existing theories, models, and frameworks, allowing exploration and critical appraisal of assumptions, gaps, and applications. Second, theory analysis helps assess the need for further development, either by refining existing theories for new contexts or creating new ones for unexplored concepts and relationships. By systematically examining SDM theories in oral healthcare, new theoretical constructs can be identified to advance knowledge and understanding within the discipline [24]. Analyses of SDM theories, conceptual models and frameworks in healthcare have been conducted in various disciplinary contexts [25, 26, 27, 28]. To our knowledge, there has yet to be a review or synthesis of SDM theories, conceptual models, or frameworks focused solely on oral healthcare. The overall aim of this study was to appraise the quality of existing theories, conceptual models and theoretical frameworks about SDM for oral healthcare practices.

## 2 Methods

### 2.1 Study Design and Reporting Guidelines

We conducted a theory analysis of theories, frameworks and conceptual models by using a systematic search and then applying Walker and Avant's six‐step approach [25]. The six main steps for theory analysis include: (a) identify the origins; (b) examine meaning; (c) determine logical adequacy; (d) determine generalisability; (e) appraise parsimony; and (f) determine testability or whether the theory is supported by empirical data (see Table 1). As there are no reporting guidelines for theory analysis methods, we reported this study following a modified version of the Preferred Reporting Items for Systematic Reviews and Meta‐analyses (PRISMA) reporting guidelines [28].

**TABLE 1:** Walker & Avant's six theory analysis steps [25].

Steps	Elements
Origins	Country of development Authors' discipline Time of development Target domains (practice, research, education) Motivation for development Development process (source of knowledge and methodological approach)
Meaning	Concepts Relationships between concepts Underlying assumptions Schematic representation Target audience Clinical context
Logical adequacy	Deductive or inductive Predictions and hypothesis provided Logical fallacies
Generalisability	Broad or narrow focus Applicability to other clinical contexts Breadth of content
Parsimony	Clarity of language Exhaustivity, clarity and simplicity
Testability and usefulness in practice	Authors discussion on how the model can be tested If a theory can be tested (generate hypothesis) Or supported with empirical data (study design, results, interventions/tools to use with the model)

### 2.2 Data Sources

We developed the search strategy with guidance from two librarians (AD, EB) (see Table 2). The search terms were selected according to three main concepts: (a) oral or dental healthcare, (b) shared decision‐making and (c) theory. Each concept was expanded to include synonyms and partial words. There were no limitations regarding geography or date of publication. The searches were performed in Medline and translated for Scopus and CINAHL on June 30, 2023.

**TABLE 2:** Documentation for search in Medline (OVID).

Steps	Search term	Number of articles found
1	Exp oral Health	20,249
2	Dental Hygienists/	5,900
3	Dentistry/or dental care/	58,139
4	Dental health services/or dental care	26,660
5	(oral health or oral care or Dental Hygienist* or dentist* or dental*). ti, ab, kf	287,114
6	1 or 2 or 3 or 4 or 5	315,222
7	Decision making/or decision making, shared/	105,856
8	Patient‐Centred Care/	22,863
9	(patient centred care* or patient‐centred care* or patient focused care or patient‐focused care* or person centred care* or person‐centred care* or person focused care* or person‐focused care* or shared decision making or shared decision‐making or shared decision‐making or SDM or shared decision or decision making or decision‐making or decision‐making or decisional need or decision aid or Patient Participation or dentist‐patient relation). ti, ab, kf.	171,944
10	Patient Participation/	29,370
11	Dentist‐Patient Relations/	8,152
12	(patient activation or patient empowerment or patient engagement or patient involvement). ti, ab, kf.	8,827
13	7 or 8 or 9 or 10 or 11 or 12	279,804
14	6 and 13	8,276
15	Models, Theoretical/	162,076
16	(theoretical model* or theoretical framework* or theory framework* or theory model* or conceptual framework* or conceptual model* or dental care model* or dental care framework* or oral health model* or oral health framework* or ontology). ti, ab, kf.	84,876
17	15 or 16	240,276
18	14 and 17	83
19	Limit 18 to English language	79

*Note:* exp = exploded MeSH term; = Non exploded MeSH term; ti, ab, kf. = Title, abstract and author keywords.

* Truncation of word for alternate endings.

### 2.3 Eligibility Criteria

Eligible citations had to include a theory, conceptual model or framework developed by the authors of the article. By definition, theory, conceptual models and frameworks share the feature of offering a set of theoretical understandings of the relationships between concepts about a particular phenomenon [29]. Yet there are distinctions among them. Conceptual models are generally descriptive and refer to a simplification of a general phenomenon. Frameworks usually provide a structure or overview of differently defined concepts. Similar to conceptual models, frameworks are often accompanied by a visual representation of the relationships between concepts. Theories aim to organise the understanding and explanation of the world using concepts and indicating relationships between them. Theories can be descriptive, explanatory, predictive, or prescriptive [29, 30].

Eligible theories, conceptual models, or frameworks had to: (a) focus on oral healthcare professionals engaging patients in a decision‐making process; (b) include concepts and relational statements clearly and sufficiently described to be able to conduct the analysis; and (c) be published in an English‐language, peer‐reviewed journal. We excluded articles about the development, evaluation, or implementation of interventions for oral healthcare encounters.

### 2.4 Screening for Eligible Theories, Conceptual Models, and Frameworks

Screening was conducted in two phases. In a first phase, the first author (JPK) screened titles and abstracts to identify any articles reporting on a theory, conceptual model, or framework within the oral healthcare context. In a second phase, all authors screened the full texts of retained citations from phase one using the eligibility criteria. All authors had to agree on its inclusion for the full text to be retained. We used a forward citation search of eligible articles to determine the extent to which they had been used and evaluated.

### 2.5 Analysis and Evaluation Procedures

The first author (JPK) read the included articles several times and applied Walker & Avant's theory analysis steps [24]. We extracted key information using a standardised data extraction form that was used in prior theory analyses [25, 26, 27]. Each eligible theory, conceptual model, or framework was analysed separately, and then comparisons were made across them. To consider the theories, conceptual models, and frameworks' relevance to SDM and consistent with previous reviews [25, 26, 27, 31], we mapped them onto the core elements of SDM defined by Makoul and Clayman [13]. We iteratively discussed and refined findings as a team over many rounds. The findings presented in this article were agreed upon by all authors.

## 3 Results

The initial searches across three databases resulted in 295 articles. After duplicates were removed, there were 195 articles (see Figure 1). Of 42 full‐text articles screened, we identified two articles describing two unique conceptual models: *Shared Decision Making System Dental Restoration* (SDMS‐DR) and *Four plus One Model Habit* (4 + 1HB).

**FIGURE 1:**
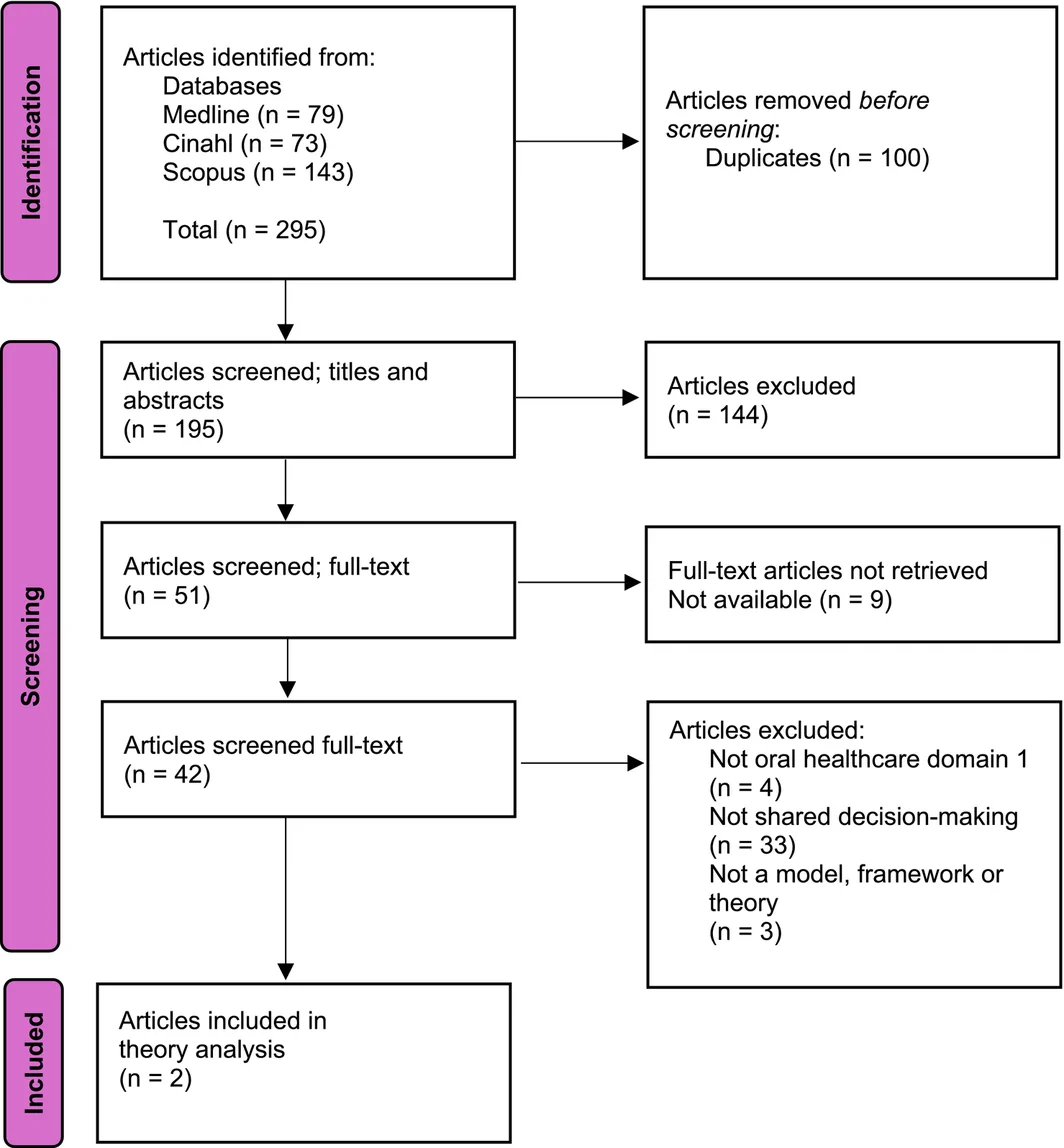
PRISMA Flow diagram.

### 3.1 Summary of Conceptual Models

The *Four plus One Model Habit* (4 + 1HB) [32] created by authors from Norway is a conceptual model specific to oral health that was an adaptation of the Four Habit Model for healthcare professionals working in various healthcare settings (not oral health) [33, 34]. According to the original Four Habit Model [33, 34], the four habits of healthcare professionals’ behaviour that impact care are: (i) investing in the beginning, (ii) eliciting the patient's perspective, (iii) demonstrating empathy, and (iv) investing in the end. The 4 + 1 HB model proposes an additional habit for oral healthcare professionals: Facilitating the patient's perceived control to permit the patient to take control of the situation and provide opportunities for the patient to contribute. This additional habit was proposed as it was recognised that patients within oral healthcare encounters were often vulnerable during assessments and treatments [32]. The authors stated that patients often regard the oral part of the body as intimate, and many patients suffer from a fear of receiving dental treatments (Figure 2).

**FIGURE 2:**
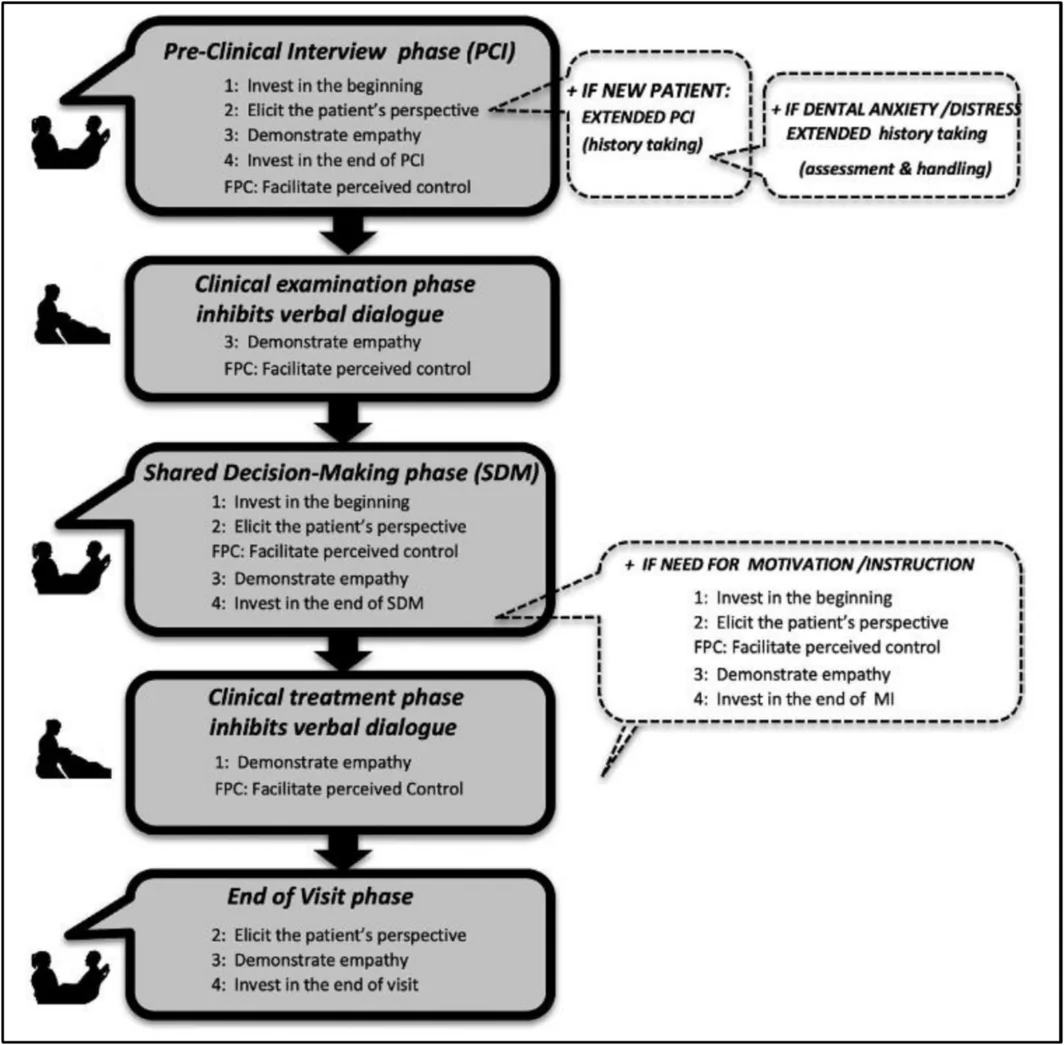
4 + 1 HB conceptual model.

The 4 + 1HB model is general, for a wide range of oral healthcare situations and decisions (see Figure 3). Despite its aim in supporting communication, the model does not describe how to enable communication to support patient engagement in evolving decisions. The authors describe decisions as amenable to revision, but without further explanation on the matter, other than to “make sure it [the agenda] is still established and understood” (p. 224). As such, the ways in which oral healthcare professionals ought to elicit patients' values and preferences are not fully described.

**FIGURE 3:**
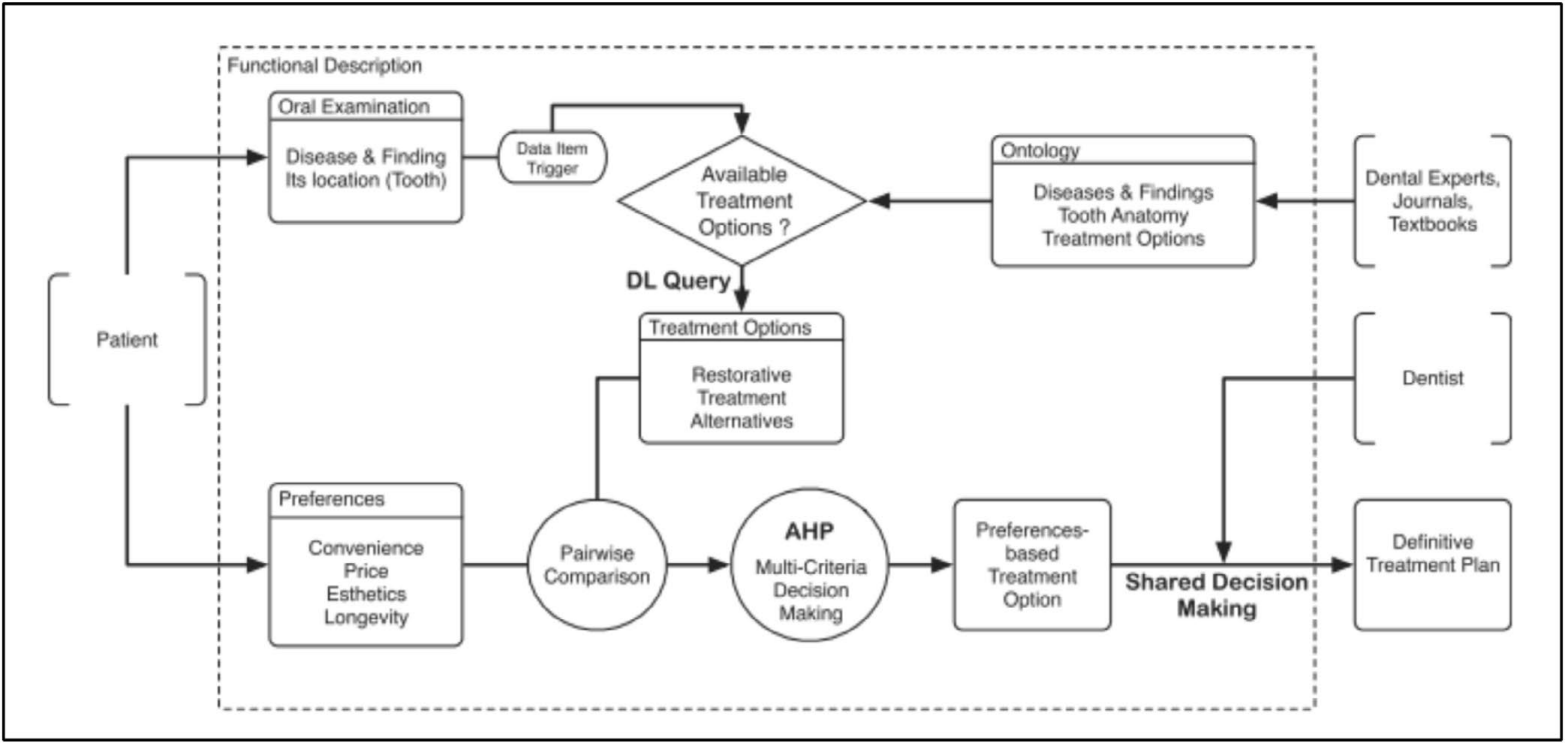
SDMS‐DR conceptual model.

The *Shared Decision Making System Dental Restoration* (*SDMS‐DR*) [35] created by authors from South Korea is a conceptual model for SDM that is specific to restorative treatment in oral healthcare (see Figure 3). In the SDMS‐DR, the analytic hierarchy process integrated within an SDM process is intended to support patients and dentists in understanding the complex decisions to be made during dental restorations and selecting a preference‐based treatment option. Using pairwise comparisons, patients are asked to rank a set of features associated with options. The SDMS‐DR addresses three main features of dental restorations: Tooth anatomy, classification of disease and findings, and restorative options. A visual representation of the conceptual model highlights how an oral examination can lead to the offering of different treatment options where patient preferences influence their suitability. SDM is described as a way to enable patients to reach an appropriate agreement on several options concerning care and treatment.

### 3.2 Theory Analysis: Comparison of Conceptual Models

We compared the 4 + 1 HB and the SDMS‐DR across each of the six theory analysis steps (Table 3).

**TABLE 3:** Summary of theory analysis results for SDM conceptual models in oral healthcare.

Elements	4 + 1 HB [32]	SDMS‐DR [35]
Origins	Norway Authors from oral healthcare and psychology Development process: Adapted from an existing model, the 4 HB model [33, 34]	South Korea Authors from oral healthcare Development process: Based on an ontology previously developed by the research group [36]
Meaning	Five phases for clinical consultation within oral healthcare consists of pre‐clinical interview, clinical examination phase inhibits verbal dialogue, shared decision‐making phase, clinical treatment phase inhibits verbal dialogue and end of visit phase. Shared decision‐making phase: ‐takes place when the clinical examination has revealed or confirmed the diagnosis, to ensure fully informed agreement on decisions. ‐The aim of the SDM phase is to ensure mutual understanding and agreement, to minimise return calls and visits, and to promote transparency, predictability, trust and collaboration. Underlying assumptions: Many patients are anxious when entering oral healthcare clinics, therefore perceived control is important for the duration of the entire clinical consultation.	Some concepts in the text are not represented in the schematic representation. Schematic, linear representation indicates: An oral examination leads to treatment options, where preferences influence patient response to pairwise comparisons in an analytic hierarchy process to achieve a preference‐based treatment option. This preferred treatment option is reviewed in a SDM encounter with the dentist to achieve a definitive treatment plan. ‐Underlying assumptions: None stated
Logical Adequacy	Logical Theoretically anchored in the four habits model [32, 33] No hypothesis presented. SDM is embedded as one phase within a larger model consisting of five phases. One‐way arrows between and within all phases indicate a non‐iterative approach to decision‐making.	Not logically adequate. Dental restorations do not treat the underlying oral disease [3] Limitations in theoretical anchoring regarding SDM. No hypothesis presented. SDM is one of the final steps in the conceptual model after patients' confirm their treatment preference
Generalisability	Wide in focus – involves oral healthcare, patients, oral healthcare professionals, and oral healthcare students. Focused on the encounter only Does not acknowledge factors influencing decision‐making at the organisational and system levels.	Narrow in focus – about restorative treatments only Involves patients and dentists Focused on the encounter only Does not acknowledge factors influencing decision‐making at the organisational and system levels.
Parsimony	Clear language Supported by a schematic representation which facilitated understanding.	Clear language Supported by a schematic representation which facilitated understanding.
Testability and usefulness in practice	No testing of the model in clinical practice or research. Phases could be used as a checklist to facilitate shared decision‐making in clinical encounters.	No testing of the conceptual model in clinical practice or research other than in a hypothetical case (presented in the article). A digital system is developed to support the SDM‐process.

*Note:* 4 + 1 HB: Four + one habits model for dental visits [32] SDM: Shared decision‐making; SDMS‐DR: Shared decision‐making system on dental restoration [35].

#### 3.2.1 Origins

The conceptual models were developed by researchers in the oral healthcare domain together with social scientists (4 + 1HB) (see Table 3) or by researchers in the oral healthcare domain only (SDMS‐DR). Both models were to enhance patient involvement in decision‐making in clinical practice and research. The motivation for developing these two conceptual models was to increase the involvement of patients in communication and decision‐making in oral healthcare. Specifically, the 4 + 1 HB aimed to support a structured SDM approach to any dental clinical consultations, and the SDMS‐DR aimed to involve the patient in dental restoration decisions. The 4 + 1HB was an adaptation of an existing model, the Four Habits model [33, 34] and the SDMS‐DR was developed deductively for use with a digital system for restorative dental treatments.

#### 3.2.2 Meaning

The two conceptual models indicate a desire to include patients' preferences in processes of decision‐making. The concepts included within the conceptual models emphasised communication, whether to “elicit the patient's perspective” (4 + 1 HB, p. 222) or “to consider the patients' preferences” (SDMS‐DR, p. 11777). The conceptual models were developed for both health professionals in oral healthcare clinical practice and for researchers interested in incorporating more person‐centred oral healthcare into their research projects. The context for SDM was limited to where oral healthcare encounters take place without consideration for general healthcare settings, interprofessional approaches, or interorganisational collaboration. Neither conceptual model considered decision‐making for individuals who are frail, with cognitive decline, or dependent on substitute decision‐makers.

#### 3.2.3 Logical Adequacy

In both conceptual models, decision‐making was considered an element of something bigger. In both the 4 + 1 HB model and the SDMS‐DR, the focus was largely on the consultation. Neither model offered predictions and hypotheses. A logical fallacy for the 4 + 1 HB model can be found within the schematic representation. The authors present the model as a checklist within oral healthcare encounters, which reduces decision‐making to a standardised, linear process rather than a dynamic and iterative approach for making shared decisions. However, in the text, the authors state that decisions might be dynamic and that the oral healthcare professionals need to acknowledge the dynamic nature. One logical fallacy for the SDMS‐DR was that in the schematic representation, the dentist is considered to hold knowledge and expertise about the condition, treatment, and their prior experience, yet the patient's expertise does not appear to be acknowledged.

#### 3.2.4 Generalisability

The 4 + 1HB is broad and can be used for any decisions in oral healthcare (4 + 1 HB), while the SDMS‐DR is treatment specific (i.e., ‘dental restorative treatment’). The 4 + 1 HB appears to be generalisable, given that it acknowledges that oral healthcare is unique due to the oral health clinical context, where many patients are anxious. As well, the 4 + 1 HB model emphasises treating patients in a holistic manner.

The SDMS‐DR does not acknowledge treatment for the underlying oral disease [3, 16], limiting its generalisability. While the 4 + 1HB referred to patients, oral healthcare professionals and oral healthcare students more broadly, the SDMS‐DR identified the patient and the dentist as the main people in the SDM encounter. Neither conceptual model acknowledged interprofessional SDM across healthcare organisations.

#### 3.2.5 Parsimony

The authors of both conceptual models presented their models using schematic representations, which facilitated understanding (see Figures 2 and 3). Concepts and the relationships between them were mostly described in a simple and clear way. Of note, while the SDMS‐DR clearly described three crucial concepts: *Tooth anatomy*, *classification of disease and findings*, and *restorative options*, they were not included in the schematic representation.

#### 3.2.6 Testability and Usefulness in Practice

Neither article described testing the conceptual models. The forward citation search of these articles did not identify any subsequent articles or studies indicating they had been evaluated. The 4 + 1 HB model presented the five phases as a checklist, which could be used to facilitate decision‐making within clinical consultations. To facilitate usefulness, the SDMS‐DR included a digital system for dentists, which could enable SDM for dentists and patients making decisions about dental restorations. However, the digital system could not be accessed by the patients; it could solely be used by dentists during the planning of treatment. It was not clear the extent to which the computer‐generated analytic hierarchy process was transferable for use in other healthcare settings.

### 3.3 Identification of Core Elements of SDM in the Conceptual Models

Both models incorporated the concept of SDM. Within the 4 + 1 HB, SDM is phase three of five, which occurs between the clinical examination phase and the clinical treatment phase. According to the SDMS‐DR, SDM occurs before a definite treatment plan is established by the dentist and the patient, and SDM is informed by the digital system's calculations.

Of the eight core elements of SDM [13], the 4 + 1HB met six elements (and 1 partially; see Table 4), and the SDMS‐DR incorporated four elements (and 1 partially). Both models encourage using SDM, explaining the clinical problem, creating choice awareness, and making a decision. Both models lacked reference to tailored information. The directionality of the SDM processes was evident in the schematic representation. The 4 + 1HB used one‐way arrows between the five phases [32] (p. 222), and the SDMS‐DR used one‐way arrows between all concepts [35] (p. 11777). Neither model indicated an iterative SDM process.

**TABLE 4:** Core elements of shared decision‐making in the conceptual models [13].

Elements	4 + 1 HB Supportive quotation (page #)	SDMS‐DR Supportive quotation (page #)
Encourages use of SDM	Yes “The SDM phase takes place as soon as clinical examination has revealed or confirmed the diagnosis, to ensure fully informed agreement on decisions” (p. 225)	Yes “The paper proposes a shared dental restorative treatment decision support system” (p. 11776)
Defines/explains the problem	Yes. Findings from “Clinical Phase, oral examination” (p. 225)	Yes From schematic model: “Disease and finding its location” and “Tooth anatomy” (p. 11777)
Creates choice awareness	Yes “Involve patient in discussion of treatment goals, options and their implications” (p. 226)	Yes “Before a patient decides on a restorative treatment, various preferences are typically taken into consideration” (p. 11777)
Encourages learning about the patient and offer preference‐based treatment options	Yes “The patient's treatment goals and expectations should be discussed, as well as his own responsibilities. Patient's preferences, requests and potential barriers should also be discussed” (p. 225)//“Provide treatment options and explain rationale. Check patient's knowledge and comprehension” (p. 226)	Partially N/A
Encourages awareness of (oral) healthcare professionals' knowledge/recommendations	Yes “Habit component in dentistry: Deliver diagnostic information” (p. 226)	Yes “The doctor is responsible for the latter comparison because the patient is not equipped to fully understand the characteristics of the restorative alternatives.” (p. 11777)
Encourages making the decision (make or explicitly defer decision)	Partially N/A	Yes “The doctor and the patient can now make a shared decision with the help of the value for the restorative alternative”. (p. 11778)
Encourages tailoring information	No N/A	No N/A
Encourages deliberation	Yes “Facilitate perceived control is therefore important during negotiation of the treatment plan” (p. 225).	No N/A

## 4 Discussion

We identified two conceptual models in the oral healthcare field that focused on engaging patients in decision‐making. The 4 + 1 HB model is broader in applicability, while the SDMS‐DR was specifically developed for dental restoration. The two conceptual models were mostly parsimonious, considered patients' values and preferences in decision‐making, and explicitly used the term *SDM*. However, they seemed to integrate the elements of SDM as a byproduct of something else, as a small part within dental consultations or within the development and use of a digital system [32, 35]. Despite not having been tested, both conceptual models offered tools to help facilitate their use in oral healthcare practices. Our findings lead to four main discussion points.

First, despite the establishment of SDM as the gold standard approach to decision‐making in healthcare more broadly [34, 37], only two conceptual models for SDM specific to oral healthcare were identified. Our findings yielded pioneering work on conceptual models in oral healthcare, indicating that it appears to be a field new to SDM. This is similar to previously published discipline‐specific SDM theory analyses. For example, three conceptual models were identified as relevant for both physiotherapy and nurses working in an interprofessional setting [27, 28]. There are other general SDM conceptual models, but to the best of our knowledge, none explicitly reported their relevance or use for oral healthcare settings, and none were found to be used in oral healthcare studies [38]. Further research is required to consider their relevance to oral healthcare, necessary adaptations, and evaluation of use in oral healthcare studies.

Second, interestingly, neither conceptual model discussed all oral healthcare team members. One was focused on dentists only [32] and the other was a general approach to decision making with dentists [33]. However, dental hygienists and dental nurses are also important members of the oral healthcare team, and dental hygienists have been considered key coordinators for integrating oral healthcare with general healthcare [39]. SDM theories that are more inclusive of all oral healthcare team members could support dental hygienists to navigate ethically complex decisions, involve patients in decision‐making, and provide evidence‐based, person‐centred care in the integrative field of oral and general healthcare [24].

Third, SDM theories, conceptual models, and frameworks could be used to establish a common ground between general healthcare and oral healthcare [24] to support professionals to be more person‐centred. This is a particularly relevant omission in oral healthcare, given that care implications and decisions are often relevant to and transfer across discipline‐specific healthcare services. Yet, our findings showed that neither conceptual model integrated interprofessional approaches nor conceptualised SDM across healthcare and oral healthcare services. Oral healthcare services are provided by interprofessional teams including dentists, dental hygienists, dental nurses, and dental assistants [40]. The 4 + 1HB model did include oral healthcare professionals more generally, whilst the SDMS‐DR were limited to dentists only. Previous research has shown that implementation of SDM requires engagement of the full interprofessional team [41], and this should be no different in oral healthcare settings. One conceptual model that does acknowledge the interprofessional team is the Interprofessional SDM Model [42], which has not been used in the oral healthcare context.

Fourth, interventions without a solid theoretical base might not include the active ingredient(s) necessary to derive benefit, nor choose the most appropriate outcomes to be evaluated [43, 44, 45, 46]. Oral health issues often involve multiple factors, including dental, medical, and behavioural aspects [9]. Additionally, oral healthcare decisions can be influenced by broader systemic factors such as access to care, insurance coverage, and public health policies [3]. This indicates that general SDM models may need customisation and modifications to fully address the unique complexities and interdisciplinary nature of oral healthcare. Therefore, this article focuses on theories, models, and frameworks specifically designed for oral healthcare. However, existing general approaches could potentially be tested in the field to ensure more holistic and equitable care. Thus, when developing and testing SDM interventions for the oral healthcare context, other well‐established SDM conceptual models and frameworks, such as the Ottawa Decision Support Framework [47] and the Three‐Talk Model [48], could be considered or adapted.

### 4.1 Strengths and Limitations

Two key strengths of this study were using Walker and Avant's [24] structured approach to analyse existing conceptual models within oral healthcare, and the use of a previously validated analysis tool [25, 26, 27]. Another strength was the collaboration with two academic librarians when creating and executing the search strategy. Although one author conducted the initial analysis, findings were iteratively developed and finalised through multiple discussions with co‐authors. We limited our search strategy to articles published in the English language only, making it difficult to know if any SDM models in oral health were published in other languages. Finally, we excluded intervention studies. It is possible that new or modified SDM theories for oral healthcare emerged from these types of articles.

## 5 Conclusion

Our theory analysis identified and appraised two conceptual models used for research studies about SDM in oral healthcare. The 4 + 1 HB conceptual model focused more generally on SDM in oral healthcare, and the SDMS‐DR was specific to decisions about restorative treatments. Both conceptual models focused on the clinical encounter where patients and oral healthcare professionals would engage in SDM without considering interprofessional, interorganisational, or intersystem level factors that influence patient involvement in SDM. Neither conceptual model has been tested. Further development or adaptation of theoretical underpinnings for patient engagement in SDM about oral healthcare is needed to promote interprofessional collaboration and ensure holistic and equitable oral healthcare. For this to happen, dental hygienists could be considered key drivers for this change.

## 6 Clinical Relevance

Scientific Rationale for Study: This study aimed to evaluate the quality of theories, conceptual models, and theoretical frameworks about SDM in oral healthcare practices.

Principal Findings: The analysis identified two conceptual models specific to oral healthcare: The SDM System on Dental Restorations (SDMS‐DR) from South Korea and the Four + one Habits Model (4 + 1HB) from Norway. Both models focus on the patient‐professional encounter.

Practical Implications: The findings highlight the need for more comprehensive and empirically tested SDM models in oral healthcare that incorporate patient expertise and broader healthcare contexts.

## Author Contributions

All authors contributed to the conception and design of the study, acquisition of data, analysis, and interpretation of the findings. All authors have provided final approval of the version to be submitted.

## Funding

This work was supported by the Health Promotion Research Funding, Region Västra Götaland, Sweden (VGFOUREG‐937918, VGFOUREG‐966932).

## Ethics Statement

The authors have nothing to report.

## Consent

The authors have nothing to report.

## Conflicts of Interest

The authors declare no conflicts of interest.

## Supporting information


**Data S1:** PRISMA_2020_checklist.

## Data Availability

The data that support the findings of this study are available from the corresponding author upon reasonable request.
